# The protective effects of electroacupuncture on intestinal barrier lesions in IBS and UC model

**DOI:** 10.1038/s41598-023-34182-z

**Published:** 2023-05-04

**Authors:** Shuqing Liu, Qin Huang, Qianhui Huang, Yuemei Wang, Sihui Li, Junmeng Wang, Qiaofeng Wu

**Affiliations:** Acupuncture and Moxibustion School, Chengdu University of Traditional Chinese Medicine, Chengdu, 610075 Sichuan People’s Republic of China

**Keywords:** Gastrointestinal diseases, Gastrointestinal diseases

## Abstract

Irritable bowel syndrome (IBS) and ulcerative colitis (UC) are two intestinal diseases with different pathological changes. Electroacupuncture (EA) at Zusanli (ST36) on both IBS and UC is widely used in clinic practice. But it is unclear whether acupuncture at one acupoint can treat two different intestinal diseases at different layers of intestinal barrier. To address this question, we explored three intestinal barrier lesions in IBS and UC mice with the aid of transcriptome data analysis and studied the efficacy of EA at ST36 on them. The transcriptome data analysis showed that both UC and IBS had disrupted intestinal barrier in various layers. And both UC and IBS had epithelial barrier lesions with reduction of ZO-1, Occludin and Claudin-1, while UC rather than IBS had the destruction of the mucus barrier with less MUC2 expression. As to the vascular barrier, UC showed a higher CD31 level and mesenteric blood flow reduction, while IBS showed a lower PV-1 level. EA at ST36 can significantly improve the above lesions of intestinal barrier of IBS and UC. Our results gave more details about the comprehensive protective effect of EA for UC and IBS. We guess the effect of acupuncture may be a kind of homeostasis regulation.

## Introduction

Irritable bowel syndrome (IBS) is a functional bowel disease characterized by abdominal pain and altered bowel habits. It is divided into the diarrhea-predominant IBS (IBS-D), the constipation-predominant IBS, or the mixed bowel habit IBS subsets according to the symptoms^1^. Ulcerative colitis (UC) is a form of inflammatory bowel disease characterized by relapsing inflammation of the colonic mucosa^2^. Although both of the two diseases are intestinal diseases, their pathogenesis and pathological characteristics are different. Among the numerous causes of disease, intestinal barrier plays a crucial role in protection against environmental threats and the resident microbiota. The dysfunction of intestinal barrier is recognized as an essential pathogenic factor in UC, and an impaired intestinal barrier function has become evident also in IBS^3,4^. In recent years, more and more studies advocate that the damage degree of intestinal tissues should be evaluated from different layers of colonic intestinal barrier (usually identified as mucus barrier, epithelial barrier and vascular barrier^5^), so as to better elucidate and screen the therapeutic targets.

Acupuncture, a traditional Chinese medical therapy, has proven efficacy for IBS, UC, postoperative gastrointestinal dysfunction, Crohn's disease, functional constipation and other digestive diseases in clinical trials^6–10^. And our previous study also found that moxibustion showed efficacy in treating drinking dextran sodium sulfate (DSS)-induced UC model mice^11^. Recent literature analysis shows that Zusanli (ST36) is commonly used in acupuncture treatment of IBS and UC^12,13^. Some studies have found that electroacupuncture can increase the level of ZO-1, Occludin in the colonic epithelial barrier of IBS rats^14,15^. Other studies showed that electroacupuncture could increase the expression of Mucins-2 (MUC2), a crucial mucin of intestinal mucosal barrier, both in IBS and UC^16^. Studies on UC mice demonstrated that electroacupuncture can alleviate the apoptosis of epithelial cells, thereby protecting the epithelial barrier and mucosal barrier^17^. Therefore, acupuncture can effectively improve the pathological changes of UC and IBS. However, it is still unclear whether acupuncture at one acupoint can treat two different intestinal diseases at different layers of colonic intestinal barrier.

In this study, we first compare the lesion of different layers of colonic intestinal barrier of IBS and UC with the aid of transcriptome data analysis. Then, we detected the specific molecular and proteins expressed on different layers before and after electroacupuncture treatment. In this way, we want to comprehensively understand the characteristics of the effects of electroacupuncture at ST36 in two different intestinal diseases.

## Results

### Transcriptomic analysis revealed that intestinal barrier in UC and IBS was disrupted to different degree in the three layers of intestinal barriers

**Figure 1:**
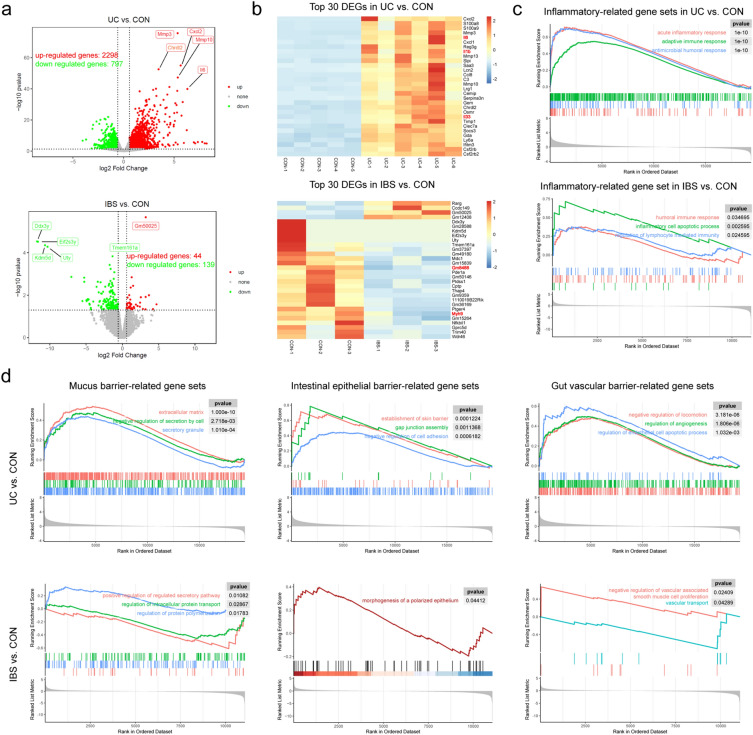
Transcriptomic analysis revealed disrupted intestinal barrier in Ulcerative colitis (UC) and irritable bowel syndrome (IBS). (**a**) Volcano plots presenting the intestinal differentially expressed genes (DEGs) between UC and CON, IBS and CON. (**b**) Heatmaps showing the top 30 differentially expressed genes. There were some inflammatory-related genes in UC vs. CON, such as *Il6*, *Il1b* and *Il33*, and some cell motility-related genes in IBS vs. CON, such as *Gm8488* and *Myh9*. (**c**) Enrichment plots from inflammatory-related gene set enrichment analysis (GSEA). (**d**) Enrichment plots from gene set enrichment analysis about intestinal mucus barrier, epithelial barrier, gut vascular barrier.

In order to explore the difference in intestinal barrier pathological changes between UC and IBS, we analyzed the colon transcriptome data of DSS-induced UC mice and the Bifidobacterium adolescentis-induced IBS mice^18,19^. The result of differentially expressed genes (DEGs) analysis showed that the intestinal changes of UC mice (3095 DEGs) were more severe than those of IBS mice (183 DEGs, Fig. 1a). Among them, most of the DEGs with significant changes in UC mice were inflammatory-related genes, such as *Il6*, *Il1b* and *Il33*, while those in IBS mice were actin-related genes that promote cell movement, such as *Gm8488* and *Myh9* (Fig. 1b). In addition, gene set enrichment analysis (GSEA) revealed that the pathways related to inflammation and immune response were enriched in UC mice and IBS mice, but it was more severe in UC mice (Fig. 1c).

Notably, the changes in three intestinal barriers of UC and IBS were not identical. The mucus barrier, which is mainly composed of the extracellular matrix and is affected by the level of cell secretion, showed high levels of extracellular matrix and secretory granules in UC mice according to GSEA analysis. However, negative regulation of secretion by cells was also observed in UC mice. In contrast, IBS mice only showed negative regulation of the secretory pathway and intracellular protein transport. Regarding the epithelial barrier, UC mice showed a high level of gap junction components and negative regulation of cell adhesion, while IBS mice showed morphological changes in polarized epithelial cells. In terms of the gut vascular barrier, UC mice showed negative regulation of locomotion and changes in angiogenesis and endothelial cell apoptosis. But the changes in IBS mice were mainly in vasoconstriction, such as negative regulation of vascular associated smooth muscle cell proliferation (Fig. 1d).

### Electroacupuncture improved colonic lesions and permeability

**Figure 2:**
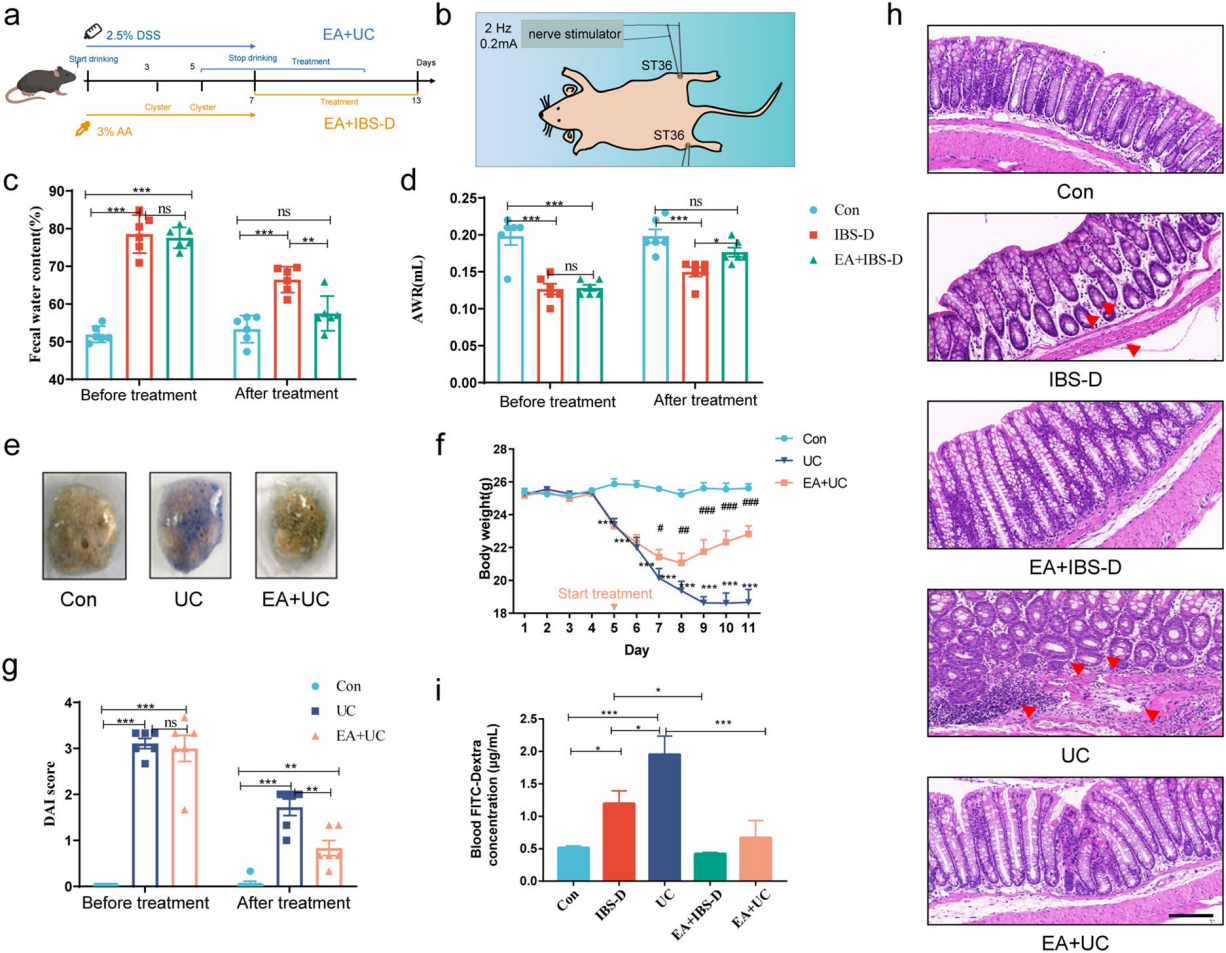
Intestinal lesions, inflammation and mucosa permeability were improved after electroacupuncture. (**a**) Diagram of modeling and treatment regimen. The mice in the electroacupuncture (EA) + UC group were treated on the fifth day of the model, and the mice in the EA + IBS group were treated after the modeling. (**b**) Schematic diagram of EA treatment at the Zusanli (ST36). Two needles were inserted into the bilateral ST36, and connected to the poles of the nerve stimulator. (**c** and **d**) The change of fecal water content and abdominal withdrawal reflex (AWR) before and after treatment in IBS experiment (n = 6). (**e**) Fecal occult blood test. (**f**) The changes of body weight in UC experiment (n = 6). (**g**) The change of disease activity index (DAI) before and after treatment in UC experiment. (**h**) Pathological changes in colon tissue (HE staining). Arrows point to disorganized glands distribution, thinned muscular layer and inflammatory cell infiltration. Scale bar, 100 μm. (**i**) Evaluation of intestinal permeability using FITC-dextran (n = 4). One-way ANOVA test with posttest *P < 0.05, **P < 0.01, ***P < 0.001, ns *P* > 0.05.

To observe the therapeutic effect of electroacupuncture at ST36 on intestinal barrier of UC and IBS, we induced UC model mice with DSS and IBS-D model mice with acetic acid and began electroacupuncture treatment on the 5th day of DSS feeding and the 7th day of acetic acid modeling (Fig. 2a and 2b, see Materials and Methods for details).

After treatment, the fecal water content and abdominal wall reflex (AWR) test on IBS-D mice, and fecal occult blood, body weight change and disease activity index (DAI) score on UC mice were used to assess the therapeutic effect of electroacupuncture. The results showed that before treatment, the fecal water content in IBS-D group and electroacupuncture + IBS-D (EA + IBS-D) was significantly higher than that in control (Con) group (*P* < 0.001), and after treatment, compared with Con group, the fecal water content in IBS-D group was still higher (*P* < 0.001), while that in EA + IBS-D group was significantly lower than that in IBS-D group (*P* < 0.01, Fig. 2c). Similarly, the visceral sensitivity of IBS-D mice measured by AWR test was significantly improved after electroacupuncture treatment (*P* < 0.05, Fig. 2d). In the UC model experiment, the fecal occult blood of UC mice was significantly relieved after electroacupuncture treatment (Fig. 2e). From the third day of treatment, the weight loss was improved (*P* < 0.05, Fig. 2f), and the DAI decreased significantly (*P* < 0.01, Fig. 2g).

In order to further verify the effect of electroacupuncture on intestinal lesions in IBS-D and UC mice, we evaluated the pathological changes of distal colon using HE staining. Results showed that in the Con group, the colonic mucosa appeared normal with neatly arranged villi and glands, and no obvious fracture or inflammatory cell infiltration in the submucosa and muscular layer. In contrast, no significant pathological changes were found in the IBS-D group, except for glandular disorganization and thinning of the muscle layer. However, in UC group, the colonic mucosa showed a blurred appearance, defects of varying sizes, scattered arrangement of glands, submucosal edema, and infiltration of a large number of inflammatory cells. After treatment, the mucosal structure of both IBS-D and UC mice improved, and the degree of mucosal inflammatory cell infiltration was reduced in UC mice (Fig. 2h).

The increased permeability of intestinal barrier is an essential factor leading to systemic inflammation in UC and lesions in IBS-D. Therefore, we used Fluorescein isothiocyanate-Dextran (FITC-Dextran) to evaluate intestinal permeability. Compared with Con group, the permeability of IBS-D group and UC group increased significantly (*P* < 0.05 and *P* < 0.001), and the permeability of UC group was higher than that of IBS-D group (*P* < 0.05). After electroacupuncture intervention, the permeability of EA + IBS-D group was lower than that of IBS-D group (*P* < 0.05), and that of EA + UC group was significantly lower than UC group (*P* < 0.001, Fig. 2i).

### Electroacupuncture recovered intestinal mucus barrier and reduced inflammation

**Figure 3:**
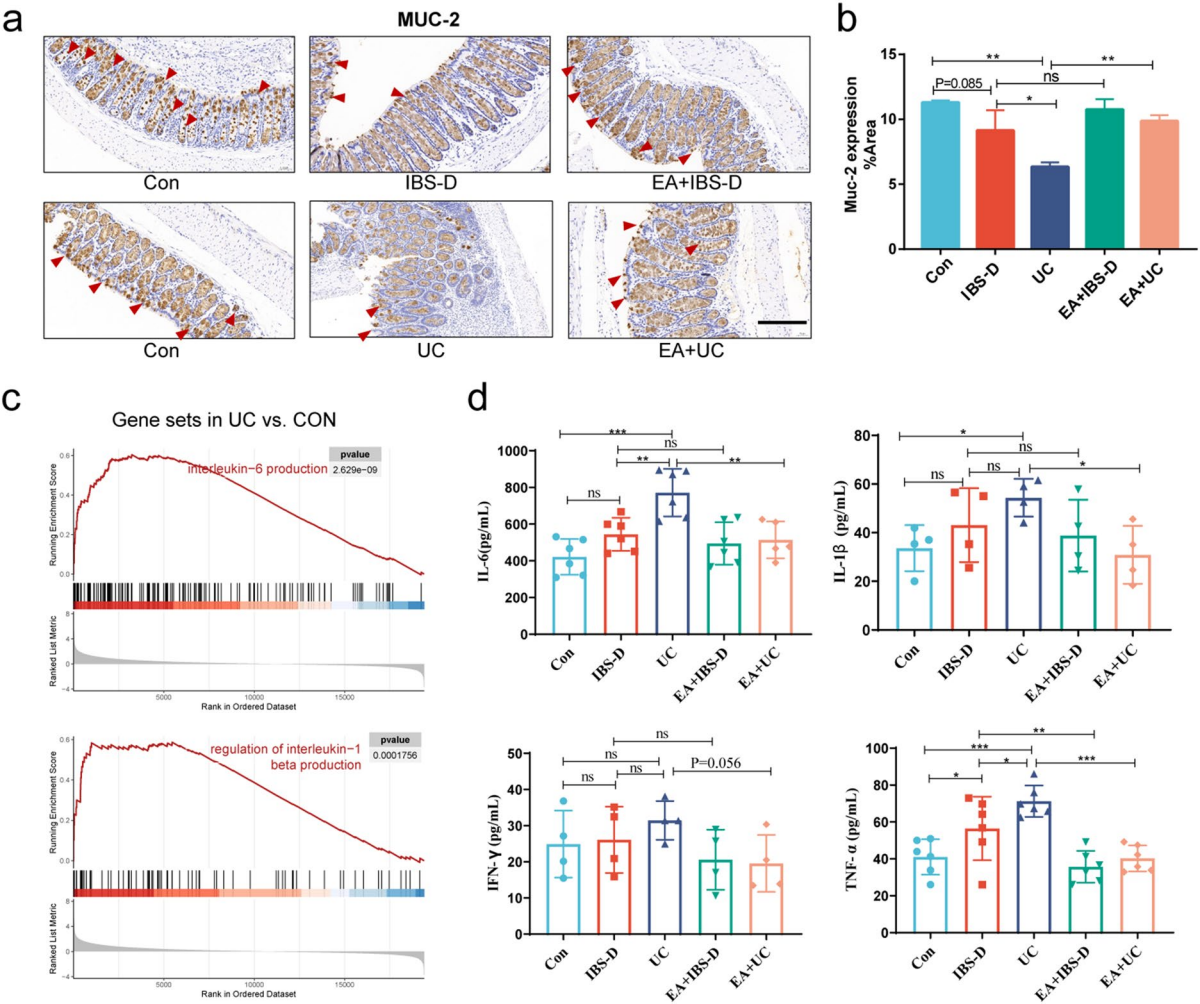
Intestinal mucus barrier was improved after electroacupuncture. (**a**) MUC-2 immunohistochemical staining. Arrows point to strong positive stain. Scale bar, 100 μm. (**b**) Barplot showed the proportion of MUC-2 positive area in each group (n = 3). (**c**) Enrichment plots from interleukin-related GSEA. (d) Enzyme linked immunosorbent assay (ELISA) analysis of serum IL-6, IL-1β, IFN-γ and TNF-α levels (n = 6). One-way ANOVA test with posttest *P < 0.05, **P < 0.01, ***P < 0.001, ns *P* > 0.05.

To systematically analyze the changes in intestinal barrier of IBS-D and UC, we explored the intestinal mucus barrier, epithelial barrier and vascular barrier, respectively. As mentioned earlier, the changes of intestinal mucus barrier of IBS-D and UC were related to the changes in cellular secretory function (Fig. 1d). To further evaluate the effect of electroacupuncture on the mucosal barrier, we used immunohistochemistry to detect the expression levels of Mucins-2 (MUC-2), which is an essential component of the mucus barrier. The results showed that compared with Con group, the expression of MUC-2 in colon of IBS-D group had no noticeable change (*P* > 0.05), but the expression of MUC-2 in colon of UC group significantly reduced (*P* < 0.01), which even was lower than that of IBS-D group (*P* < 0.05). After treatment, the expression of MUC-2 protein in intestine of EA + IBS-D group had no obvious change, but the expression of MUC-2 in colon of EA + UC group was significantly higher than that of UC group (*P* < 0.01, Fig. 3a and 3b).

Production of pro-inflammatory factors often accompanies the destruction of intestinal mucus barrier. The GSEA results also showed obvious interleukin-6 (IL-6) and interleukin-1β (IL-1β) reactions in UC (Fig. 3c) and mild inflammatory and immune responses in IBS-D, as mentioned earlier. In order to explore the effect of electroacupuncture on inflammation of UC and IBS-D, we measured the IL-6, IL-1β, interferon-γ (IFN-γ) and tumor necrosis factor-α (TNF-α) levels in serum by enzyme linked immunosorbent assay (ELISA). Compared with Con group, TNF-α levels in IBS-D mice increased significantly (*P* < 0.05), but the levels of IL-6, IL-1β and IFN-γ levels had no significant change (*P* > 0.05). Interestingly, electroacupuncture could reduce TNF-α level in IBS-D mice (*P* < 0.01), but did not have effect on IL-6, IL-1β and IFN-γ levels (*P* > 0.05). In contrast, the UC group showed elevated levels of TNF-α, IL-6, and IL-1β compared to the Con group (*P* < 0.05), with no significant change observed in IFN-γ levels. After electroacupuncture treatment, the levels of TNF-α, IL-6, and IL-1β were reduced in UC mice (*P* < 0.05, Fig. 3d).

### Electroacupuncture improved intestinal epithelial barrier

**Figure 4:**
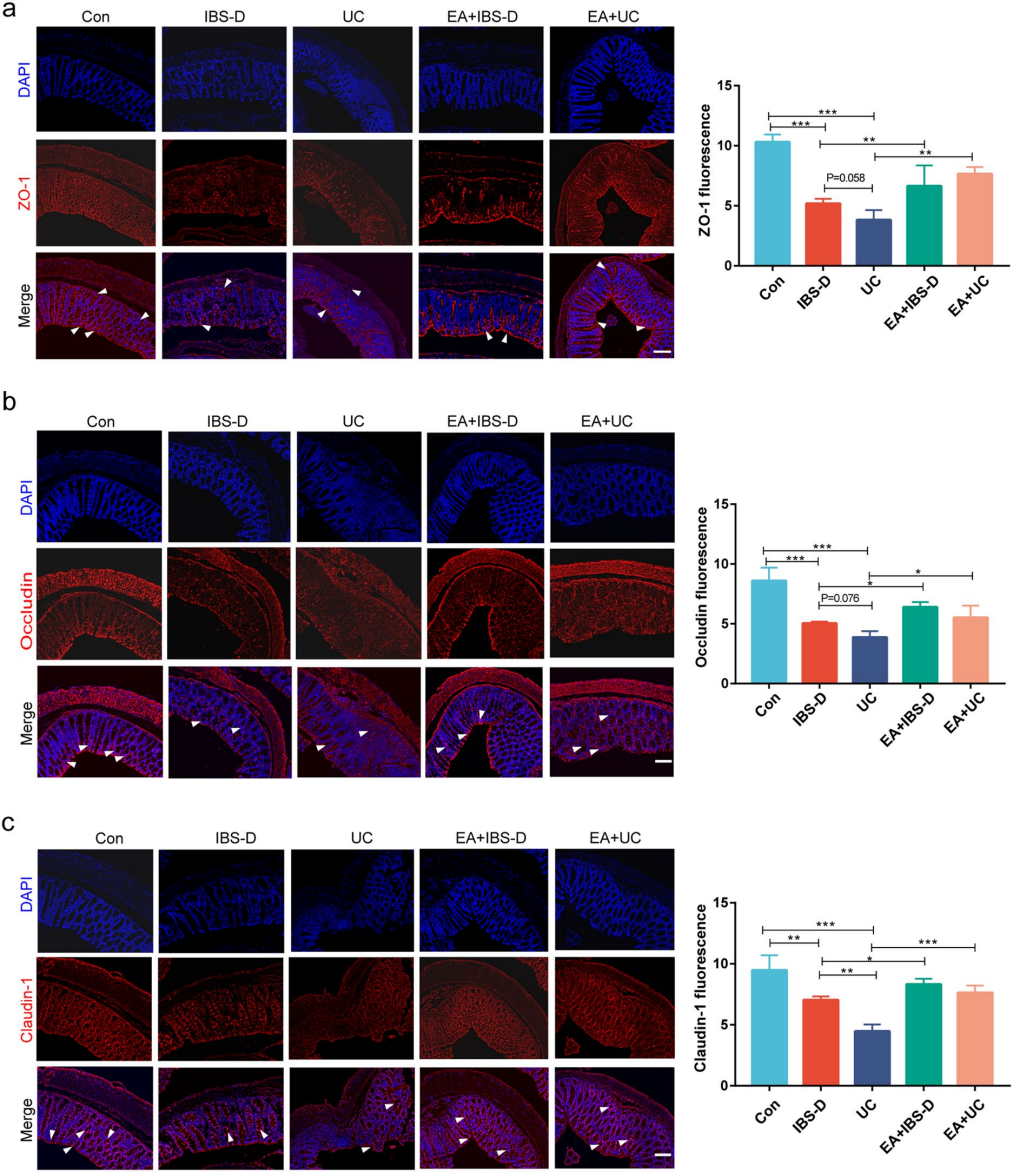
Intestinal epithelial barrier was improved after electroacupuncture. (**a**–**c**) The immunofluorescent staining of ZO-1/Occludin/Claudin-1 (red immunofluorescence) and DAPI (blue immunofluorescence). Arrows point to strong positive stain. Scale bar, 50 μm. Barplot showed the red fluorescence intensity in each group (n = 3). One-way ANOVA test with posttest *P < 0.05, **P < 0.01, ***P < 0.001.

The results of transcriptome showed that the epithelial barrier of UC and IBS-D changed significantly (Fig. 1d). To further evaluate the effect of electroacupuncture on the epithelial barrier, we used immunofluorescence to detect the expression levels of tight junction proteins that play a crucial role in the epithelial barrier. The results showed that compared with Con group, the levels of Zonula Occludens-1 (ZO-1), Occludin and Claudin-1 in colon of IBS-D group and UC group decreased (*P* < 0.01), and UC decreased more than IBS-D group (*P* = 0.058, ZO-1; *P* = 0.076, Occludin; *P* < 0.01, Claudin-1). While electroacupuncture can significantly increase the ZO-1, Occludin and Claudin-1 levels of colon in IBS-D mice and UC mice (Fig. 4a–c).

### Electroacupuncture improved gut vascular barrier and promoted mesenteric blood flow

**Figure 5:**
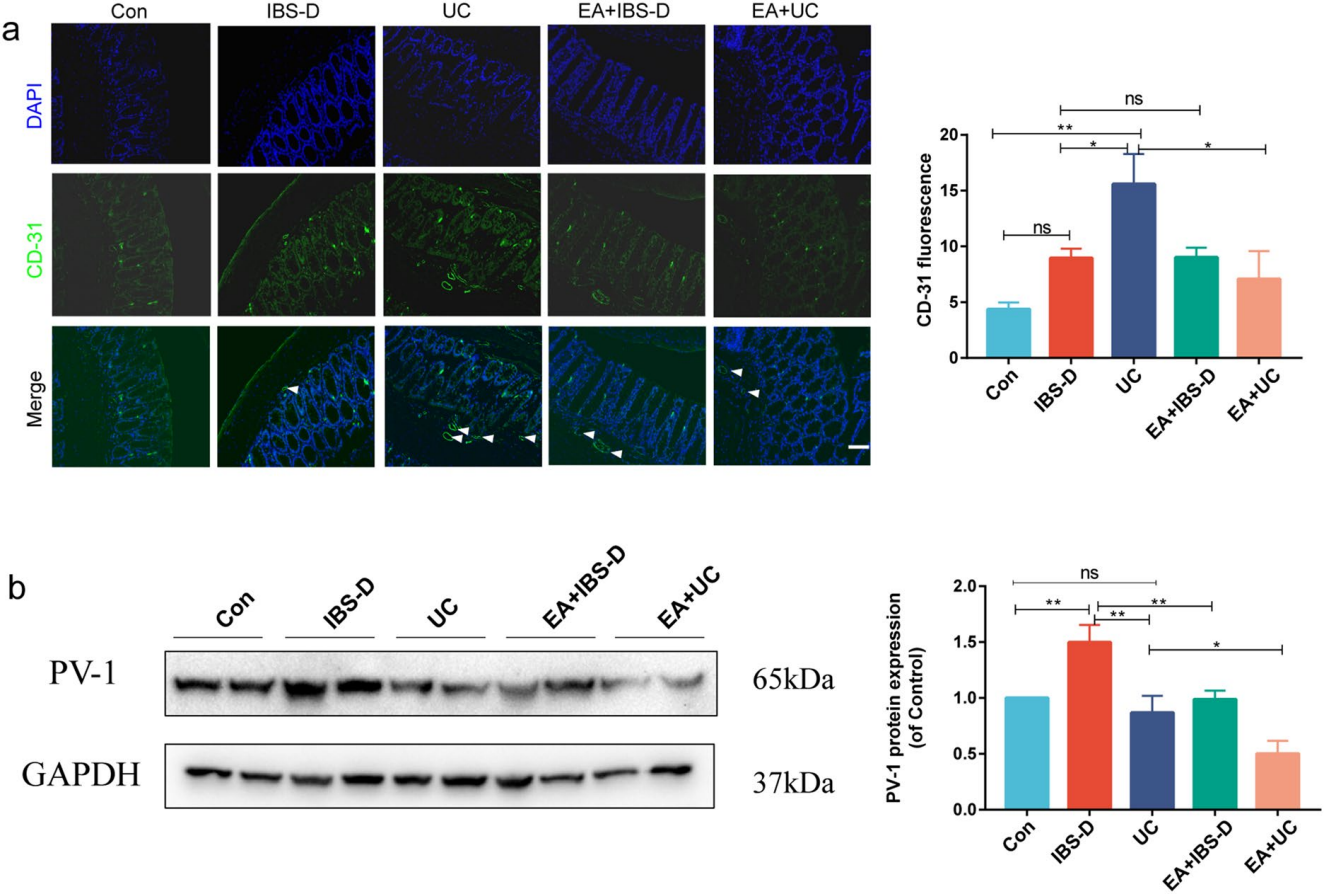
Gut vascular barrier was improved after electroacupuncture. (**a**) The immunofluorescent staining of CD-31 (green immunofluorescence) and DAPI (blue immunofluorescence). Arrows point to strong positive stain. Scale bar, 50 μm. Barplot showed the green fluorescence intensity in each group (n = 3). (**b**) Western blot analysis of PV-1 expression levels. Barplot showed the quantitative evaluation of the western blot results (n = 5). One-way ANOVA test with posttest *P < 0.05, **P < 0.01, ns *P* > 0.05.

Previously, we found that the vessels of IBS-D and UC mice may have abnormal vasoconstriction, endothelial cell proliferation, apoptosis and abnormal permeability (Fig. 1d). Therefore, we detected the expression level of vascular marker Platelet endothelial cell adhesion molecule-1 (CD-31) and vascular permeability index Plasma membrane vesicle-associated protein-1 (PV-1), and used laser speckle technique to monitor mesenteric blood flow. The immunofluorescence results showed that compared with Con group, IBS-D mice had no obvious change in CD-31 level (*P* > 0.05), but UC mice had the significant higher level of CD-31 (*P* < 0.01). Electroacupuncture could only reduce the CD-31 level of UC mice (*P* < 0.05), but not the CD-31 level of IBS-D mice (Fig. 5a). The result of Western blot showed that the level of PV-1 in IBS-D mice was significantly higher than that in the Con group (*P* < 0.01), but there was no significant change in UC mice (*P* > 0.05). Interestingly, electroacupuncture not only decreased the level of PV-1 in IBS-D mice (*P* < 0.01), but also decreased the level of PV-1 in UC mice (*P* < 0.05, Fig. 5b, Supplementary figure).

**Figure 6:**
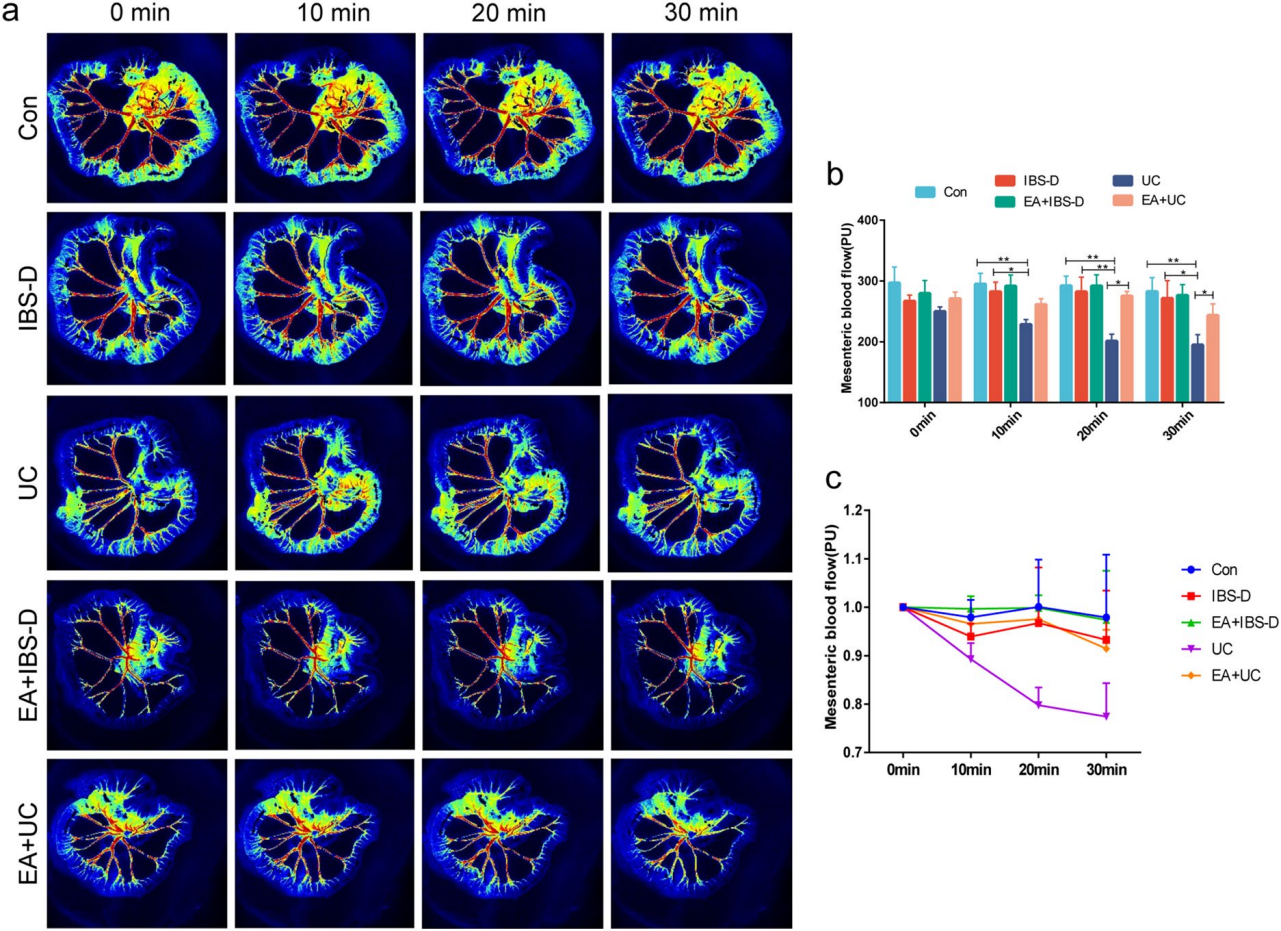
Mesenteric blood flow was improved after electroacupuncture. (**a**) Mesenteric blood flow was monitored using two-dimensional laser speckle imaging techniques (n = 6). (**b**) Relative mesenteric blood flow in each group. (**c**) Relative mesenteric blood flow with normalization in each group. One-way ANOVA test with posttest *P < 0.05, **P < 0.01.

The results of laser speckle showed that compared with Con group, the relative mesenteric blood flow of IBS-D group did not change significantly, while that of UC group decreased significantly (at 10 min, 20 min, 30 min, all *P* < 0.01). The relative mesenteric blood flow in EA + UC group was higher than that in UC group (*P* < 0.05 in 20 min and 30 min), but there was no significant change (*P* > 0.05) in EA + IBS-D group compared with IBS-D group (Fig. 6a and 6b). After data normalization, it was found that the change of Con group was small, and the relative mesenteric blood flow of IBS-D and UC groups was lower than that of Con group, with UC group showing the most significant decrease. Electroacupuncture has a limited effect on the improvement of IBS-D, but has a great improvement in UC (Fig. 6c).

## Discussions

### The pathological changes in three layers intestinal barriers of IBS and UC are slightly different

Among the three layers intestinal barriers, the mucus barrier is the first line of defense against microbes and harmful substances, which mainly consists of MUC2, water, and inorganic salts^20^. It has been found that the level of MUC2 in colon tissue of UC patients was decreased^21^ and the colon of mice knocked out of MUC2 had no mucus cover and developed colitis or colon cancer in a short period time^22^. Consistent with our results, it was reported that there was no significant change in the number of MUC2 in IBS rats, but the level of O-glycosylation, which affected the ability of MUC2 polymerization, decreased^23^. The results of our transcriptome analysis suggest that the change in the secretion or polymerization of MUC2 may be caused by the decrease in the secretion level of gut goblet cells (Fig. 1d). The epithelial barrier is a monolayer cellular barrier that separates the intestinal lumen from the outside. Tight junctions between epithelial cells are essential components to maintain the epithelial barrier, including occludins, claudins, tricellulin, cingulin, zonula occludens and junctional adhesion molecules^24^. Our findings reveal that the colonic tight junction-related proteins are significantly reduced in both IBS-D and UC mice, indicating severe damage to the epithelial barrier in these diseases, consistent with previous research^25,26^. Furthermore, inflammatory cytokines, such as IFN-γ, TNF-α, IL-1β and IL-17, have been shown to increase intestinal permeability by changing the expression of tight junction proteins or myosin light chain kinase, which regulates tight junction structure and paracellular permeability through myosin II-mediated light chain phosphorylation^27^. The TNF-α, which was elevated in the serum of IBS-D mice in our study, was also found to impair the tight junction function, induce epithelial cell apoptosis, and ultimately compromise the intestinal barrier permeability^28,29^. Gut vascular barrier is mainly formed by the association of vascular endothelial cells, pericytes and intestinal glial cells. It can prevent microorganisms and antigens from translocation to the circulatory system and affect other organs^30^. PV-1 is associated with the caveolae of fenestrated microvascular endothelial cells and is a marker of vascular barrier leakage^31,32^. Our results show that compared with UC, PV-1 can be used as a pathological indicator of the IBS-D gut vascular barrier (Fig. 4b). Fecal occult blood in UC is a direct consequence of the severe destruction of its vascular barrier. In addition, GSEA and CD-31 level of UC mice showed a high intestinal angiogenesis level (Fig. 1d and 5a), which were considered to be associated with inflammatory immune response^33^. However, the high level of angiogenesis did not promote intestinal perfusion (Fig. 6). This is believed to be related to the discontinuity and uneven distribution of newly formed capillaries in UC^34^.

### Electroacupuncture can improve the intestinal barrier damages of IBS and UC, indicating that the role of EA may be a steady-state regulation

Previous studies have indicated that UC presents more severe symptoms compared to IBS^35^. This study utilized public data and animal experiments to analyze the intestinal barriers of IBS and UC, demonstrating that both diseases induce damage to the epithelial and vascular barriers, while UC also affects the mucus barrier. Several studies showed that electroacupuncture may have a positive impact on intestinal barrier by regulating various molecular pathways. For instance, electroacupuncture has been shown to increase the intestinal ZO-1 and Occludin expression, downregulate the submucosal EGC-derived GSNO expressions^36^ and reduce the number of intestinal mucosal mast cells^15^ in IBS model. In addition, electroacupuncture has been found to inhibit the apoptosis of epithelial cells^17^ and enhance the balance of Treg/TH17 axis^37^ in UC models, which are believed to contribute to reducing inflammation and protecting the intestinal mucosal barriers. The current study showed that electroacupuncture at the ST36 acupoint significantly enhanced mucosal secretion in the intestinal barrier of UC mice, improved the expression levels of tight junction proteins in the epithelial barrier of both UC and IBS mice, reduced vascular leakage in UC mice, and decreased vascular proliferation in UC and IBS mice. These findings suggest that electroacupuncture can ameliorate intestinal barrier damage in both UC and IBS, with the extent of improvement depending on the severity of the disease.

Generally speaking, different symptoms or different pathological changes require tailored medication or specific treatments. However, in traditional Chinese medicine, the same acupoints are often used for treating various disease, such as ST36 is usually used for treating various gastro-intestinal diseases^12,13^. Until now, the mechanisms and reasons are not clear yet. Our results confirm again that electroacupuncture at ST36 is beneficial to both UC and IBS-D and this benefit seems to be similar. In modern medicine, this phenomenon is similar to the concept of steady-state regulation or homeostasis regulation. Therefore, we speculate that the effect of electroacupuncture may be a kind of homeostasis regulation.

Nowadays, many studies show that the mechanism of electroacupuncture affecting viscera by stimulating body surface acupoints is intimately associated with neuronal, humoral and paracrine pathways^38–40^. Among them, the autonomic nervous pathway was considered as one of the main pathways that can active or reduce the intestinal motility^41,42^. It is well known that the regulation of autonomic nerves plays a role in maintaining homeostasis. Therefore, elucidating the mechanism and relationship of electroacupuncture with the autonomic nerve will be future research direction in this field.

## Conclusion

To sum up, with the help of transcriptome analysis, this study elucidated the similarities and differences between IBS and UC in mucus barrier, epithelial barrier and vascular barrier. We found that EA at ST36 can reduce the lesion in corresponding layers of intestinal barriers of UC and IBS. Our results provide details for understanding that EA can treat intestinal disease, as well as deducing that the effect of electroacupuncture may be a kind of homeostasis regulation.

## Methods

### Acquisition and analysis of RNA-seq data

The RNA-seq data was acquired from the Gene Expression Omnibus (GEO) public database (GEO Accession GSE22307 and GSE215048; http://www.ncbi.nlm.nih.gov/geo/)^18,19^. The differential expression analysis was performed using EdgeR (Fold Change > 1.5 and *P* < 0.05) and the GSEA enrichment was performed using Clusterprofiler. The gent sets in GSEA derived from Gene ontology (GO).

### Animals

All experimental procedures were approved by the Animal Care and Use Committee of Chengdu University of Traditional Chinese Medicine (ethics number: 2019-04). All methods were performed in accordance with the relevant guidelines and regulations, as well as in accordance with the ARRIVE guidelines 2.0^43^. All experimental mice were purchased from Gempharmatech model animal research Co. Ltd. [license number: SCXK (chuan) 2020-034], male, 8-weeks-old and weighed 25.0 ± 2 g. Mice were housed in a vivarium environment under a 12 h light–dark cycle (humidity 50–65%, temperature 20 ± 2 °C) and had free access to diet and drinking water. After one week’s adaptation, the mice were randomly divided into 5 groups: the control (Con) group, IBS-D group, UC group, electroacupuncture + IBS-D (EA + IBS-D) group, EA + UC group.

The modeling methods of IBS-D mice and UC mice were same as the previous studies^25,44^. IBS-D model establishment: mice in IBS-D group and EA + IBS-D group were instilled of 0.1 mL of 3% acetic acid at 4 cm proximal to the anus for 10 s after being lightly anaesthetized with ether. Then, 0.1 mL of phosphate-buffered saline was instilled to dilute the acetic acid, flush the colon, and held upside down for 1 min. The above experiments were implemented once on the 3rd and 5th day of the week, for a total of 3 times. The success of the model was judged according to the fecal water content and the abdominal withdrawal reflex. UC model establishment: Drinking dextran sodium sulfate (DSS, 43 kDa, MP Biomedicals) water induced UC model mice. At the 5th day of DSS administration, the successful colitis-induced mice were checked and confirmed by fecal occult blood and the pathological morphology of the gut. The administration of DSS water to mice would last for 7 consecutive days totally. The success of the model was judged according to the fecal occult blood test (benzidine method) and the disease activity index. The mice in Con group were maintained without any treatment (Fig. 2a).

### Electroacupuncture treatment

In the UC group, as with our previous treatment, treatment began on the fifth day of modeling, which is considered to be a schedule to reduce the mortality of UC mice^44^ (Fig. 2a). The treatments to IBS-D mice were performed after modeling. Acupoints of ST36 were used for intervention. The location of the ST36 were determined according to Government Channel and Points Standard GB12346-90 of China and “The Veterinary Acupuncture of China”. Two needles were inserted into the bilateral ST36, one deep and one shallow, and connected to the poles of the nerve stimulator (Hans-200). Acupoint nerve stimulator to provide a sparse dense wave at a frequency of 2 Hz and 0.2 mA for 30 min each day and the period of care lasted for 7 days. The non-electroacupuncture treatment group used the same restraint method but did not receive electroacupuncture stimulation.

### Sample collection

After 7 days treatment, all mice were anesthetized with 1% pentobarbital sodium (3 ml/kg). For enzyme-linked immunosorbent assay, serum samples were obtained by centrifugation of the blood samples. For immunohistochemistry and immunofluorescence, colon tissues were removed, cut open, cleaned with precooling normal saline, and immersed and fixed in 4% paraformaldehyde solution. For western blot, colon tissues were separated and kept frozen at − 80 °C. For HE staining, colon tissues were paraffin-embedded, dewaxed, rehydrated, and stained with HE.

### Fecal water content

Feces were collected before and after IBS-D modeling and weighed before and after drying. The fecal water content was calculated using the following calculation formula: [wet weight (g) − dry weight (g)]/wet weight (g) × 100%.

### Abdominal withdrawal reflex (AWR)

Briefly, mice were anesthetized with isoflurane (1.5%-2.0%) and fixed in a prone position. Then the balloon was inserted through the anus and placed in the 2 cm position from the anus of mice. The tube, connected to the balloon and a syringe, was fixed with the mice tail. After the mice adapted for 30 min, the saline (0.1 ml, 0.9%) in the syringe was injected into the balloon, at the same time, the reaction of the mice was observed. The amount of saline was recorded at the pain threshold of 3 according to the AWR score standard. AWR score standard: 0 (normal behavior), 1 (slight head movement without abdominal response), 2 (contraction of abdominal muscles), 3 (lifting of abdominal wall), 4 (body arching and lifting of pelvic structures).

### Disease activity index (DAI)

The DAI score was calculated as [body weight loss score + stool consistency score + fecal occult blood score]/3. The body weight loss score ranged from 0 to 4: 0 = stable, 1 = mild loss (1% < lost < 5%), 2 = moderate loss (5% < lost < 10%), 3 = severe loss (10% < lost < 15%), and 4 = the worst loss (lost > 15%). The stool consistency score ranged from 0 to 4: 0 = normal, 2 = soft, and 4 = liquid. Fecal occult blood score ranged from 0 to 4: 0 = negative, 2 = positive, and 4 = strongly positive.

### Evaluation of intestinal permeability

Intestinal permeability was assessed using the FITC-dextran permeability assay. Briefly, mice were given a gavage of FITC-dextran (600 mg per 1 kg body weight). After 2 h, whole blood was collected, and serum was isolated. Equal volume of PBS was used to diluted serum and tested in duplicate. The concentration of FITC-dextran was determined using a fluorometer with dilutions of FITC-dextran in PBS as a standard curve.

### Immunohistochemistry

After routine dewaxing and antigen reparation, the sections were sealed with 3% bovine serum albumin. Then sections were incubated with mouse MUC2 (Cat No.27675-1-AP, Proteintech) antibody (1:200) at 4 °C overnights, washed with PBS three times, incubated with DAB chromogen for 2 h at 37 °C, and counterstained with hematoxylin for 1 min.

### Enzyme linked immunosorbent assay (ELISA)

The ELISA for mouse serum IL-6, IL-1β, IFN-γ and TNF-α levels was were performed using commercial kits from Elabscience and according to the manufacturer’s instructions.

### Immunofluorescence

After routine dewaxing and antigen reparation, the sections were sealed with 3% bovine serum albumin, and incubated with Occludin (Cat No.66378-1-Ig, Proteintech) or Claudin-1 (Cat No.13050-1-AP, Proteintech) or ZO-1 (Cat No.21773-1-AP, Proteintech), or CD-31 (Cat No. NB100-1642, Novusbio) antibody (1:200) at 4 °C overnights. Then sections were washed with PBS three times and incubated with secondary antibody (Alexa Fluor 488, cy3, Proteintech, 1:200) for 2 h at 37 °C. Photographs were taken using an immunofluorescent microscope (Nikon, Japan) and analyzed relative %area positive expression by software ImageJ.

### Western blot

Shortly, 20 μL protein aliquots from the supernatant of the tissue of the colon were run on SDS-PAGE (10%). Proteins were then transferred to 0.22 μm PVDF membranes (Millipore). Then membranes were blocked bull serum albumin (5%) in TBST for 1 h, incubated with PV-1 (Cat No.14452-1-AP, Proteintech) antibody at 4 °C overnight, and incubated with GAPDH (Cat No. GB12002, Servicebio, 1:3000) for 2 h at room temperature. Protein bands were revealed by ECL developing solution (Jiyi Nanjing) and chemiluminescence signals were detected by Bio-Rad ChemiDoc XRS (Bio-Rad). Protein band intensity was measured and quantified by ImageJ software.

### Laser speckle flowmetry

Total mesenteric blood flow was also measured using a laser speckle contrast imager (RFLS III, RWD Life Science). Five groups of mice were measured after electroacupuncture treatment. Mice were anesthetized with 1% sodium pentobarbital and placed in a supine position on a heating pad (37 ± 0.2 °C). The abdomen of the mice was cleaned, cut along the sagittal plane and covered with holed gauze. Then the mesentery was gently placed on the gauze, and 37 °C saline was dripped. Blood flow of 3 to 4 random regions of interest, was selected and measured using the onboard software (RFLSI III). The relative blood flow velocity was recorded every 10 min for a total of 30 min.

### Statistical analysis

Data are presented as the mean ± SD. Normality was tested with Shapiro–Wilk test. The weight and laser speckle data were analyzed by one-way repeated measures ANOVA. One-way ANOVA and Least-Significant Difference (LSD) were used to determine differences between groups in other data. All data analysis was conducted using SPSS26.0 software (SPSS Inc., Chicago, USA) and GraphPad Prism 8 (GraphPad Prism Software Inc., San Diego, USA). *P* < 0.05 was considered statistically significant.

### Ethics declarations

All experimental procedures were approved by the Animal Care and Use Committee of Chengdu University of Traditional Chinese Medicine (ethics number: 2019-04). All experimental mice were purchased from Gempharmatech model animal research Co. Ltd. [license number: SCXK (chuan) 2020-034].

## Supplementary Information

Supplementary Figures.

## Data Availability

The RNA-seq data was acquired from the Gene Expression Omnibus (GEO) public database (GEO Accession GSE22307 and GSE215048; http://www.ncbi.nlm.nih.gov/geo/).
