# Supplementary figures and images for: The protective effects of electroacupuncture on intestinal barrier lesions in IBS and UC model
Source: Sci Rep. 2023 May 4;13:7276. doi: 10.1038/s41598-023-34182-z (PMC10160055; doi:10.1038/s41598-023-34182-z)

# Western Blot of PV-1 and GAPDH

No.1

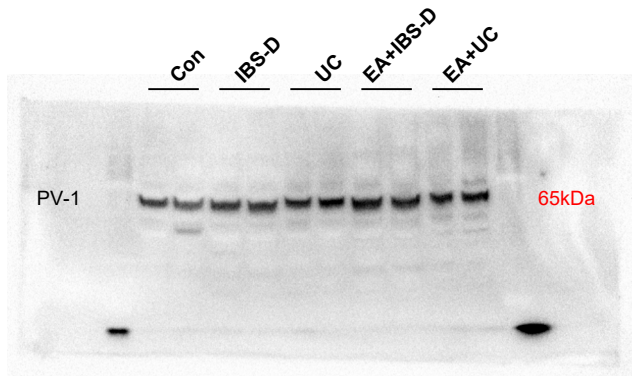

No.2

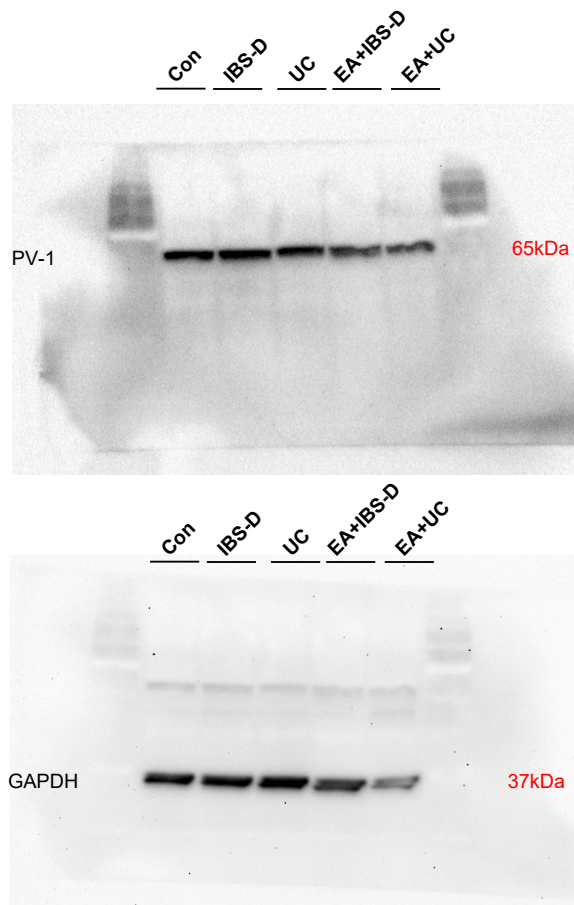

No.3

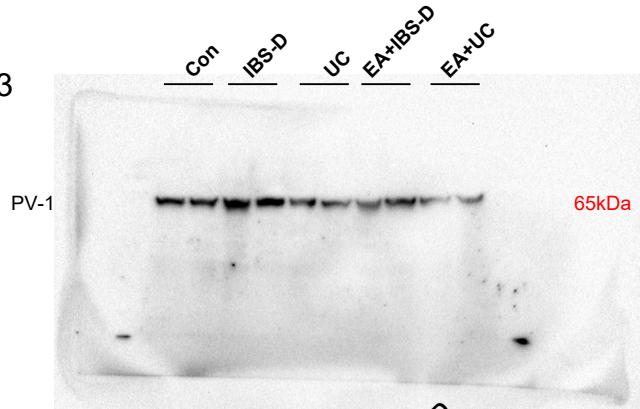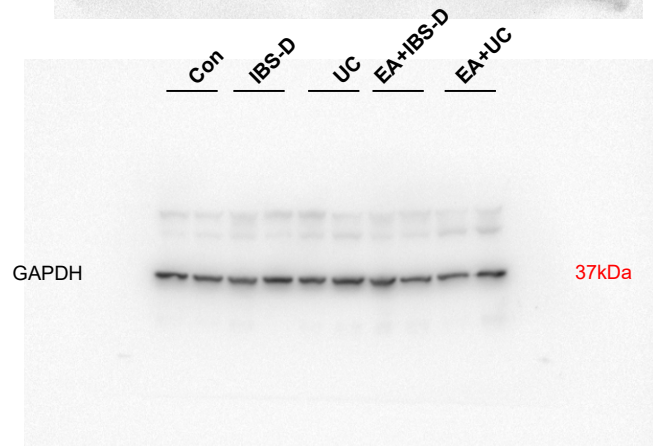

No.4

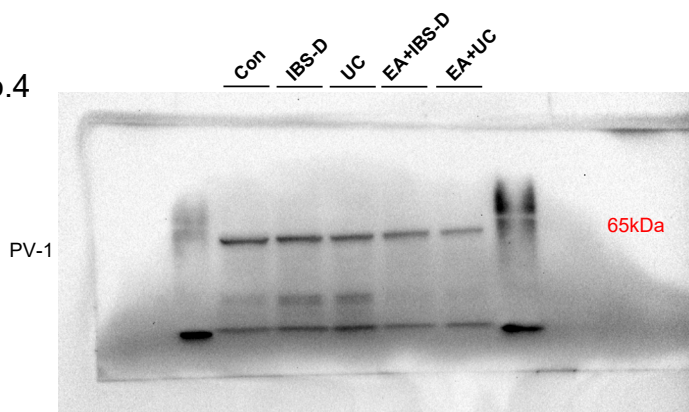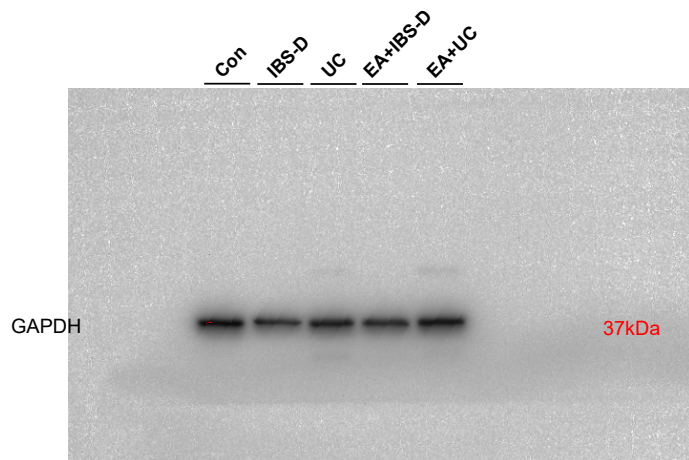

No.5

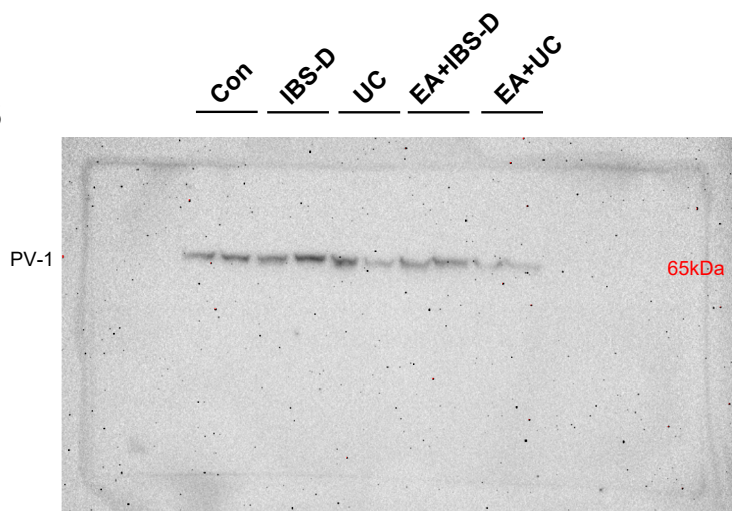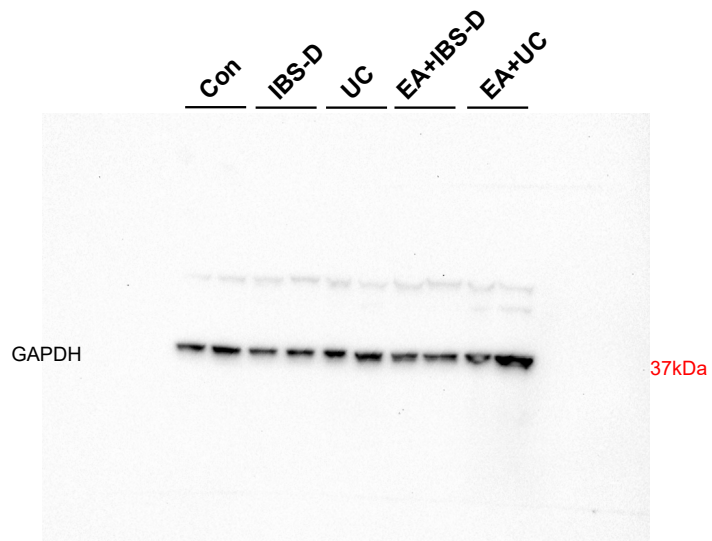

Supplement: Supplementary file 1 — Supplementary Figures. [file 41598_2023_34182_MOESM1_ESM.pdf]
